# LFchimera protects HeLa cells from invasion by *Yersinia* spp. in vitro

**DOI:** 10.1007/s10534-018-0136-0

**Published:** 2018-08-22

**Authors:** Tjitske Sijbrandij, Antoon J. Ligtenberg, Kamran Nazmi, Petra A. M. van den Keijbus, Enno C. I. Veerman, Jan G. M. Bolscher, Floris J. Bikker

**Affiliations:** Department of Oral Biochemistry, Academic Centre for Dentistry Amsterdam, University of Amsterdam and VU University Amsterdam, Gustav Mahlerlaan 3004 1081 LA Amsterdam, The Netherlands

**Keywords:** Antimicrobial peptide, Biowarfare simulants, Cellular adhesion and invasion, Lactoferrin, LFchimera

## Abstract

*Yersinia pestis* is the causative agent of plague. As adequate antibiotic treatment falls short and currently no effective vaccine is available, alternative therapeutic strategies are needed. In order to contribute to solving this problem we investigated the therapeutic potential of the peptide construct LFchimera against the safer-to-handle *Y. pestis* simulants *Yersinia enterocolitica* and *Yersinia pseudotuberculosis* in vitro. LFchimera is a heterodimeric peptide construct mimicking two antimicrobial domains of bovine lactoferrin, i.e. lactoferrampin and lactoferricin. LFchimera has been shown to be a potent antimicrobial peptide against a variety of bacteria in vitro and in vivo. Also *Y. enterocolitica* and *Y. pseudotuberculosis* have been shown to be susceptible for LFchimera in vitro. As *Yersiniae* spp. adhere to and invade host cells upon infection, we here investigated the effects of LFchimera on these processes. It was found that LFchimera has the capacity to inhibit host-cell invasion by *Yersiniae* spp. in vitro. This effect appeared to be host-cell mediated, not bacteria-mediated. Furthermore it was found that exposure of human HeLa epithelial cells to both LFchimera and the bacterial strains evoked a pro-inflammatory cytokine release from the cells in vitro.

## Introduction

*Yersinia pestis* is the causative agent of plague. Its strategy for transmission relies on the colonization of rat fleas. Once inside the human host, *Y. pestis* can cause bubonic, pneumonic, and septicaemic plague with mortality rates approaching 100% in absence of antibiotic treatment. The Centers for Disease Control and Prevention (CDC) classifies *Y. pestis* as a category A biological warfare agent (BWA) (Jansen et al. 2014; Rotz et al. 2002). In order to overcome several safety, security and logistical drawbacks in biomedical investigations on *Y. pestis* research is often performed using it safer simulants *Yersinia enterocolitica* and *Yersinia pseudotuberculosis* (Adducci et al. 2016; Bikker et al. 2006; Dawson and Liu 2008; Kaman et al. 2011; Sijbrandij et al. 2017). *Y. enterocolitica* and *Y. pseudotuberculosis* can cause yersiniosis, an infectious disease which generally lasts for one to three weeks (Galindo et al. 2011). Infection can occur by the consumption of undercooked meat, unpasteurized milk or contaminated water. Common symptoms include fever, abdominal pain, and in children diarrhea, which can be bloody in severe cases. Complications are rare, and may include skin rash, joint pains, ileitis, erythema nodosum, septicemia, and acute arthritis (Bancerz-Kisiel and Szweda 2015).

Since most of the symptoms caused by *Y. enterocolitica* and *Y. pseudotuberculosis* are self-limiting, specific antibiotic treatment is generally not needed, especially in relative mild cases. For example, patients with dehydration from gastroenteritis are given supportive therapy, including treatment aimed at replacing fluids. Antibiotics are indicated, however, for those patients who develop more severe infections, such as septicemia, or who develop infections at specific sites, such as bone. But, in case of plague-caused by *Y. pestis*-antibiotic treatment must be started immediately without laboratory confirmation to avoid the risk of transmission and mortality. Antibiotics generally used for treatment include aminoglycosides and trimethoprim-sulfamethoxazole. Also third-generation cephalosporins, tetracyclines and fluoroquinolones can be applied, yet with limited use in children. Although these antibiotics are still used, resistance is emerging (Guiyoule et al. 1997, 2001; Hernandez et al. 2003; Hinnebusch et al. 2002). Besides, as currently no effective and licensed vaccine is available for the prevention of plague (Oyston and Williamson 2013; Verma and Tuteja 2016) alternative strategies to develop therapies against *Yersinia* related infections are needed.

LFchimera is a heterodimeric peptide construct designed to mimic two antimicrobial domains, Lactoferricin (LFcin) and Lactoferrampin (LFampin), which are present in the N1-domain of bovine lactoferrin (bLF) and are responsible for its antimicrobial activity in situ (Bolscher et al. 2009, 2012). LFchimera has been shown to be a potent antimicrobial peptide against a variety of bacteria (Bolscher et al. 2009) in vitro and in vivo, including *Staphylococcus aureus* (Flores-Villaseñor et al. 2010), *Escherichia coli* O157:H7 (Flores-Villaseñor et al. 2012a), *Streptococcus pneumoniae* (León-Sicairos et al. 2014) and *Burkholderia pseudomallei* (Kanthawong et al. 2009; Puknun et al. 2013, 2016), the latter being classified by the CDC as a category B biological warfare agent (BWA) (Rotz et al. 2002). Also *Y. enterocolitica* and *Y. pseudotuberculosis* have been shown to be susceptible to LFchimera (Sijbrandij et al. 2017). Overall, the bactericidal activity of the LFchimera is related to membrane disturbing effects, i.e. membrane permeabilization and depolarization.

During infection, *Yersiniae* first adhere to host cells, followed by invasion. So, hypothetically, inhibition of cell adhesion and invasion would contribute to the therapeutic effect of the LF peptides, on top of their antimicrobial activity. Therefore, the aim of the presented study was to map the effects of bovine LF (bLF) derived peptides on adhesion and invasion of *Y. enterocolitica* and *Y. pseudotuberculosi*s. For this study it was chosen to use human HeLa epithelial cells as the in vitro model system, as Hela cells or HeLa-derived cells were used in literature before for *Yersinia* studies (Dersch and Isberg 1999; Di Biase et al. 2004; Schulte et al. 1996, 2000).

## Materials and methods

### bLF and LF derived peptides

bLF (20% iron saturated) was kindly provided by DMV International (Veghel, The Netherlands). LF derived peptides (Table 1) were synthesized by solid phase peptide synthesis using Fmoc chemistry with a Siro II synthesizer (Biotage, Uppsala, Sweden) according to the manufacturer’s protocol. Purification by Reverse Phase-HPLC was conducted as described previously (Bolscher et al. 2009). Identity of the peptides was confirmed by mass spectrometry (Bruker Daltonik GMBH, Bremen, Germany) and molar concentrations were calculated based on their weight.

**Table 1:** Sequences and characteristics of the peptides investigated

Peptide^a^	Primary structure	Charge	MBC (μM)^b^	
			*Y. e.*	*Y. p.*
LFampin265–284	DLIWKLLSKAQEKFGKNKSR	+ 4	6.3	ND
LFcin17–30	FKCRRWQWRMKKLG	+ 6	0.8	ND
LFchimera^c^		+ 12	0.2	1.6
LFampin265–284 and LFcin17–30	DLIWKLLSKAQEKFGKNKSR and FKCRRWQWRMKKLG	+ 10	0.8	ND

*Y. e.*: *Y. enterocolitica*, *Y. p.*: *Y. pseudotuberculosis*

^a^The purity of the peptides was at least 95% and the authenticity of the peptides was confirmed by MALDI-TOF mass spectrometry

^b^Minimal bactericidal concentration (MBC), in 1 mM phosphate buffer (from Sijbrandij et al. 2017). ND: not detected up to 50 µM peptide

^c^A single C-terminal lysine amide (**K**) was substituted at the α- and ε-amino groups with the two peptides via the C-terminal site, while leaving the two N-termini as free ends

### Bacterial strains, cell line and culture conditions

*Yersinia enterocolitica* (DSM 4780) and *Y. pseudotuberculosis* (DSM 8992) were cultured overnight aerobically in trypticase soy broth (TSB) medium at 30 °C and colony forming units (CFU) were determined on trypticase soy agar (TSA) as described earlier (Sijbrandij et al. 2017). Human HeLa epithelial cells were grown in RPMI1640 medium (RPMI, Life Technologies, Carlsbad, USA) supplemented with 10% fetal bovine serum (HyClone, South Logan, USA) and 1% penicillin/streptomycin/amphotericin B (Sigma-Aldrich, St. Louis, USA) at 37 °C with 5% CO_2_.

### Bactericidal activity of LF peptides

The killing activities of the LF peptides against the *Yersinia* spp. were determined as described previously with the exception that the incubation was performed in RPMI (Sijbrandij et al. 2017). In this way conditions were the same as for cytotoxicity determinations and adhesion and invasion assays (see below). Briefly, bacterial cells were washed three times and approximately 2 × 10^6^ CFU/ml were re-suspended in RPMI medium. The bacterial suspension was then added to an equal volume of the tested agents (0.1–50 µM final concentration). A bacterial suspension in RPMI without peptide served as a control. Following incubation at 37 °C for 60 min, the mixture was serially diluted in a physiological concentration of saline and plated in triplicate on TSA. After 24 h of incubation at 37 °C colonies were counted. A bactericidal effect was defined as a ≥ 3 log_10_ reduction in CFU/ml compared with the initial inoculum.

### Cytotoxicity of bLF and LF peptides on HeLa cells

Cytotoxicity of bLF and the LF peptides toward HeLa cells was analyzed by monitoring mitochondrial activity. For this, approximately 5 × 10^4^ HeLa cells were seeded in a 96-well plate and cultured serum-free for 16 h. Cells were washed twice with PBS. A serial dilution of bLF and each peptide was made in PBS (0–25 µM). Cells we were incubated at 37 °C under 5% CO_2_ for 1 h. Maximal cytotoxicity was induced by adding 0.1% Triton X-100 (Sigma-Aldrich, St. Louis, MO, USA). Cells were washed twice with PBS and incubated in PBS containing 0.5 mg/ml 3-(4,5-dimethylthiazol-2-yl)-2,5-diphenyltetrazolium bromide (MTT, ThermoFisher Scientific, Bleiswijk, the Netherlands) for 2 h and washed again. The MTT-crystals that were precipitated in the cells were resuspended in 100% dimethylsulfoxide (DMSO, Sigma-Aldrich). Absorption was measured at 570 nm with 630 nm for background correction using a Multiscan FC microplate photometer (ThermoFisher Scientific,). In parallel, cytotoxic effects of bLF and the peptides on Hela cells were analyzed by measuring the lactate dehydrogenase (LDH) release using an LDH-cytotoxicity kit (Abcam, Cambridge, UK) according to the manufacturer’s instructions (Sijbrandij et al. 2017). The experiments were performed in duplicate and repeated three times.

### Effects of LF peptides on bacterial adhesion and invasion

The effect of LF peptides on adhesion and invasion of the *Yersinia* spp. were carried out essentially as described previously (Wang et al. 2008; Tan et al. 2017). Briefly, HeLa cells were plated at a density of 1 × 10^5^ cells in 24-wells plates (Greiner, Recklinghausen, Germany). After attachment the LF peptides were added at concentrations up to 3.1 μM. After 1 h, the unbound peptides were removed by washing three times using PBS. Fresh stocks of bacteria were harvested by centrifugation at 10,000×*g* for 10 min, washed twice in PBS and resuspended in PBS to a density of 5 × 10^8^ cells/ml. Next, bacteria were added to the HeLa cells at a multiplicity of infection (MOI) of 100 CFU/cell. Subsequently, the 24-wells culture plates were centrifuged at 300×*g* for 10 min to promote contact between bacteria and HeLa cells and then incubated at 37 °C with 5% CO_2_ for 2 h and washed three times with PBS to remove unadhered bacteria. To determine the number of cell-associated bacteria, this solutions was serially diluted in a physiological concentration of saline and plated in triplicate on TSA and colonies were counted after 24 h of incubation at 37 °C as described previously (Sijbrandij et al. 2017).

To determine the number of intracellular bacteria, extracellular bacteria were killed by treatment of 100 μg/ml gentamicin in fresh medium during 2 h. The cells were further cultured at 37 °C with 5% CO_2_ for 4 h, washed three times with PBS and lysed by the addition of 0.1% Triton X-100 at 37 °C with 5% CO_2_ for 20 min. Again, this solutions was serially diluted in a physiological concentration of saline and plated in triplicate on TSA and colonies were counted after 24 h of incubation at 37 °C as described above. The number of cell-associated bacteria subtracted by the number of intracellular bacteria was used as measure for adhesion.

As control for treatment timing, LFchimera up to 3.1 μM was added for 1 h post infection, before or after gentamicin treatment in the above described protocol. Furthermore, the effect of preincubation of bacteria or preincubation of HeLa cells with 3.1 μM LFchimera at 37 °C for 1 h were tested similarly.

### Effect of LFchimera on cytokine release

The effect of LFchimera on the release of cytokine IL-6 and chemokine IL-8 from HeLa cells upon infection with the *Yersina* spp. were analyzed using in essence the experimental set-up as described above. Briefly, HeLa cells were plated at a density of 2 × 10^5^ cells in 24-wells plates. Fresh stocks of bacteria were harvested by centrifugation at 10,000×*g* for 10 min, washed 3 × in PBS and resuspended in serum-free medium to a density of 5 × 10^8^ cells/ml. Next, the HeLa cells were washed twice with PBS and medium was added with 0, 1.6 or 3.1 µM LFchimera. The cells were incubated for 1 h washed twice with PBS and bacteria were added to the HeLa cells at a multiplicity of infection (MOI) of 100 CFU/cell and centrifuged at 300×*g* for 10 min to promote contact between bacteria and HeLa cells. After incubated for 2 h at 37 °C, 5% CO_2_ the 24-wells culture plates were washed three times with PBS to remove unadhered bacteria and further incubated in 0.5 ml serum free medium for 24 h. Subsequently, the concentrations of IL-6 and IL-8 were determined in 100 µl samples from the 24-wells culture plates after remove the bacteria by centrifuged at 10,000×*g* for 5 min, using the PeliKine compact human IL-6 and IL-8 ELISA kits according to the manufacturer’s protocol (Sanquin, Amsterdam, The Netherlands).

### Statistical analysis

Statistical analysis was performed using a one-way ANOVA and posthoc Bonferroni test using SPSS Statistics for Windows version 20.0 (IBM Corp, Armonk, NY, USA). *P* values < 0.05 were considered statistical significant.

## Results

### Bactericidal activity of LF peptides

**Fig. 1:**
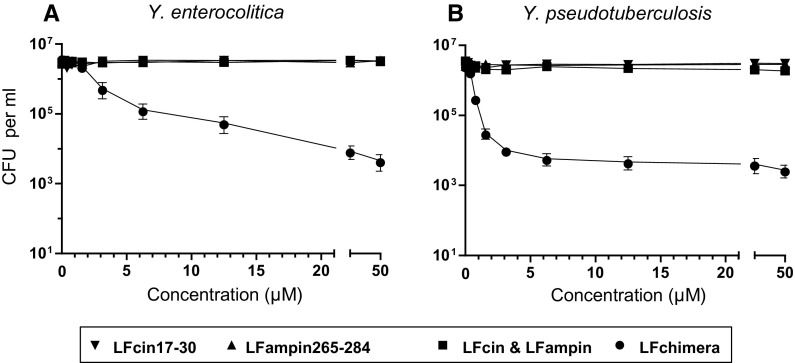
Bactericidal activity of bLF-peptides on *Y. enterocolitica* (**a**) and Y. *pseudotuberculosis* (**b**) determined in RPMI cell culture medium after an incubation of 1 h. Bactericidal effect was defined as a ^10^log[reduction] > 3 in viability. Data are shown as mean values ± SEM from three independent experiments carried out in triplicate

Before exploring the potential effect of the LF peptides on adhesion and invasion of the *Yersinia* spp., anti-bacterial activity of these peptides in HeLa cell culture medium was measured, to ensure that subsequent experiments are performed at sub-bactericidal levels. Under these conditions both LFcin17–30 and LFampin265–284 as well as the combination of the two peptides had no bactericidal effect on the *Yersinia* spp. (Fig. 1). Only the LFchimera, exhibited inhibitory activities in a dose response manner. LFchimera at a concentration 3.1 µM caused ^10^log = 1 reduction of *Y. enterocolitica* increasing to almost 3 log_10_ reduction at higher concentrations up to 50 µM. *Y. pseudotuberculosis* was a little more sensitive as 3.1 µM LFchimera induced a ^10^log > 2 reduction in CFU count, being close to the maximal effect of less than 3 log_10_ with 50 µM (Fig. 1). However, in contrast to the bactericidal activity at low ionic strength (Table 1), the anti-bacterial effect of LFchimera in cell culture medium remained below the ^10^log = 3 reduction in CFU counts, used as cut-off value for bactericidal effect (Sijbrandij et al. 2017). Therefore the concentration of 3.1 µM LFchimera in HeLa cell culture medium was considered to be a sub-lethal dose towards the *Yersinia* spp. used.

### Cytotoxicity of bLF and the LF peptides

**Fig. 2:**
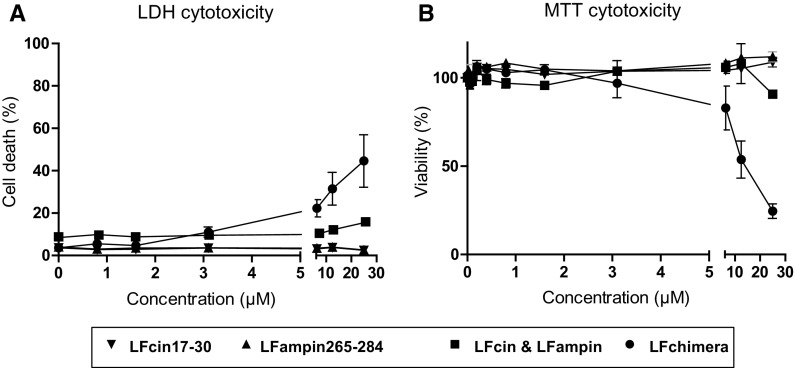
HeLa cell cytotoxicity of bLF-peptides determined in RPMI medium after an incubation of 1 h: LDH-leakage as measure for cell death (**a**) and MTT assay as measure for viability (**b**). Data are shown as mean values ± SEM from three independent experiments carried out in triplicate

To prevent using cytotoxic levels of the LF peptides in subsequent experiments, cytotoxicity toward HeLa cells was determined by measuring leakage of LDH as well as by monitoring mitochondrial activity (Fig. 2). In both assays LFcin17–30 and LFampin265–284 as well as the combination showed no or only minor effect up to 25 µM concentrations. The first sign of any cytotoxic effect was found at LFchimera concentrations of 6.1 µM. Therefore peptide concentrations up to 3.1 µM were considered to be not cytotoxic and were used to analyze the effect on the adhesion and invasion of the *Yersinia* spp. bLF did not show any cytotoxic effect up to 25 μM (data not shown).

### Adhesion and invasion

**Fig. 3:**
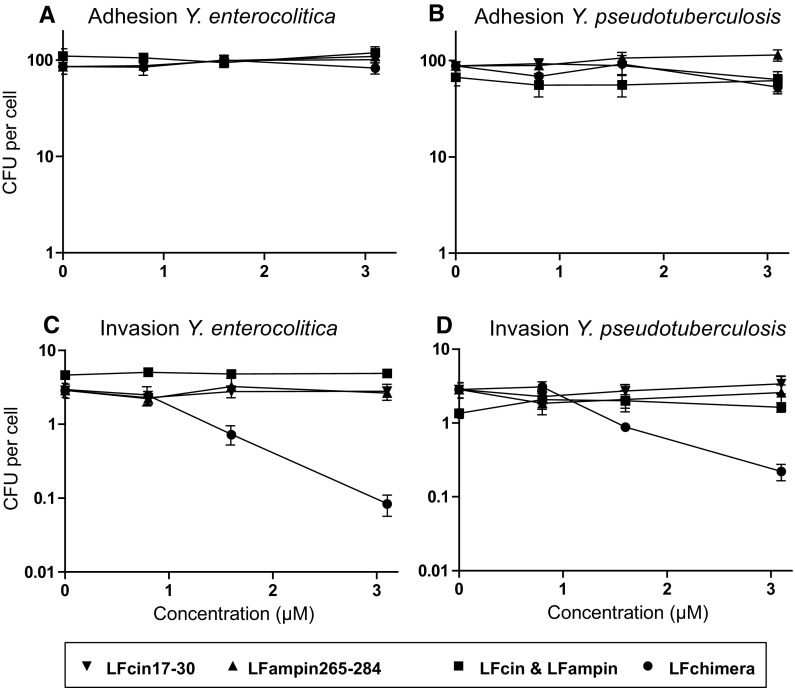
Effects of bLF-peptides on adhesion and invasion of *Y. enterocolitica* (**a**), *Y. pseudotuberculosis* (**b**), *Y. enterocolitica* (**c**), and *Y. pseudotuberculosis* (**d**) on HeLa epithelial cells treated for 1 h with non-lethal concentrations of peptides. Data are shown as mean values ± SEM from three independent experiments carried out in triplicate

None of the LF peptides at a concentration up to 3.1 µM showed any effect on the adherence of both *Y. enterocolitica* and *Y. pseudotuberculosis* to the HeLa cells (Fig. 3). Strikingly, invasion of both *Yersinia* spp. was exclusively inhibited by LFchimera at concentrations as low as 1.6 µM. At a concentration of 3.1 µM LFchimera, the invasion of *Y. enterocolitica* was inhibited about twice as much as *Y. pseudotuberculosis* (30-fold and 15-fold respectively, Fig. 3). In contrast, LFcin, LFampin and the combination of the two peptides had no effect on the capacity of the *Yersinia* spp. to invade the HeLa cells.

**Fig. 4:**
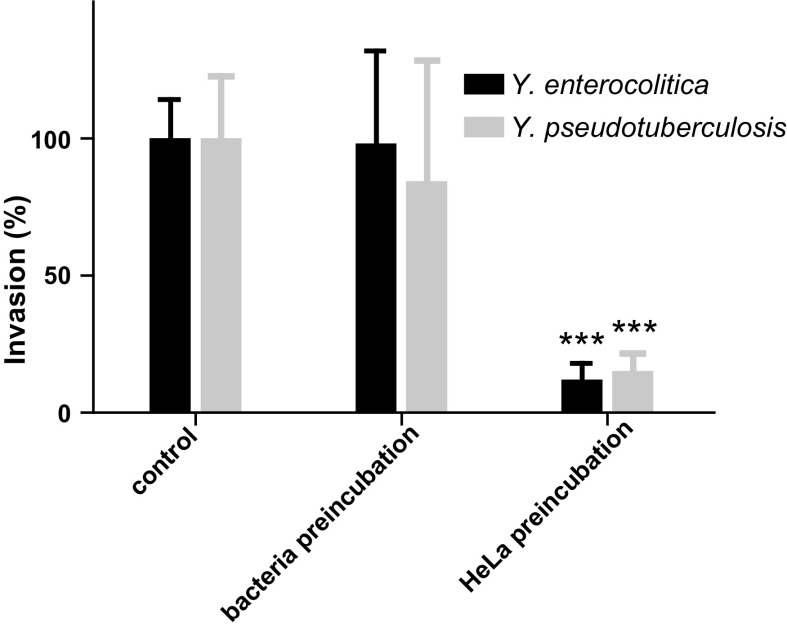
Inhibition of invasion by 3.1 µM LFchimera after preincubated with either bacteria or HeLa cells for 1 h. Invasion is presented as percentage of the controls without LFchimera. Data are shown as mean values ± SD from three independent experiments carried out in triplicate. Asterisk indicates statistical significant differences versus control incubations (***P < 0.0005)

Next, we determined whether the inhibition was mediated through an effect on the *Yersinia* spp. or on the HeLa cells. Pretreatment of the *Yersinia* spp. with 3.1 µM LFchimera had no effect on the invasion whereas pretreatment of the HeLa cells significantly reduced the invasion capacity of both *Yersinia* spp. by circa 80% of the level of invasion without LFchimera (Fig. 4). Control experiments in which LFchimera was added post infection, before or after gentamicin treatment indicated that the LFchimera did not made the cells permeable to gentamicin nor killed the bacteria intracellularly (data not shown).

### Cytokine and chemokine release

**Fig. 5:**
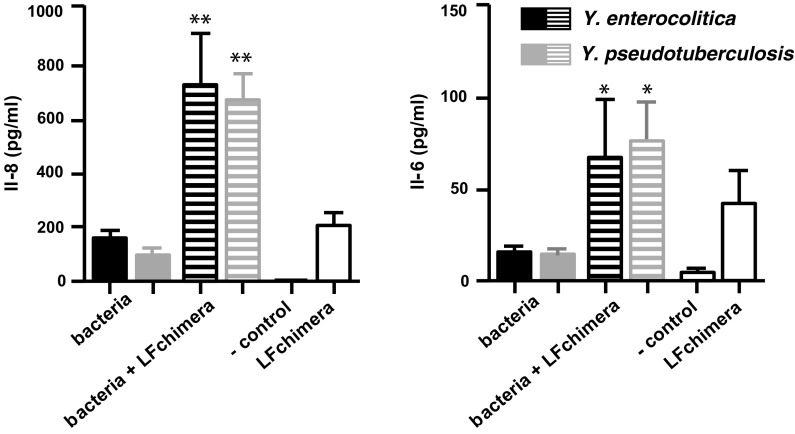
Effects of 3.1 µM LFchimera on IL-8 (**a**) and IL-6 (**b**) expression upon infection with and without *Y. enterocolitica* and *Y. pseudotuberculosis*. Data are shown as mean values ± SD from three independent experiments carried out in triplicate. Asterisk indicates statistical significant differences versus the incubations without LFchimera (*P < 0.05; **P < 0.005)

Because LFchimera inhibited invasion of the *Yersinia* spp. through an effect on the target cells, we investigated whether the HeLa cells released inflammatory mediators, such as IL-6 and IL-8, upon infection with bacteria. Indeed, an almost fourfold increase in the release of IL-8 by *Yersinia*-infected HeLa cells was found in the presence of 3.1 µM LFchimera (Fig. 5). The release of IL-6 was also increased, be it that LFchimera also stimulated the IL-6 production of uninfected HeLa cells to some extent (Fig. 5).

## Discussion

In the present paper we show and characterize the effects of LFchimera on the capacity of *Y. enterocolitica* and *Y. pseudotuberculosis* to adhere to-, and invade HeLa cells. Previously it was found that in 1 mM potassium phosphate buffer (PPB), LFchimera exhibited bactericidal activity at concentrations as low as 0.2 and 1.6 µM towards the *Yersinia* spp., respectively (Table 1; Sijbrandij et al. 2017). In contrast, in RPMI medium, used to culture HeLa cells, the anti-*Yersinia* activity of LFchimera was only found at much higher concentrations (Fig. 1). Though LFchimera had different antimicrobial effect on the individual species, at concentrations that are sub-lethal and not cytotoxic in RPMI medium, i.e. up to 3.1 μM, LFchimera inhibited the capacity of the *Yersinia* spp. to invade HeLa cells (Fig. 1). Inhibition of invasion could be induced by pre-incubation of the HeLa cells but not by pre-incubation of the bacteria. As reference, the native bLF showed no bactericidal and cytotoxic activity in vitro up to 50 µM and 25 μM, respectively. Therefore, the study subsequently focused on the LF peptides only.

In line with this study invasion of *Escherichia coli* (EHEC) O157:H7 was found to be inhibited by LFchimera in vivo. BALB/c mice inoculated intragastrically with EHEC O157:H7 showed chronic intestinal infection with the pathogen that persisted over 6 days and resulted in a high mortality rate (90%). LFchimera could significantly decrease this mortality rate by 50% (Flores-Villaseñor et al. 2012a). Moreover, it was found that LFchimera inhibited invasion of enteropathogenic *E. coli* to Hep-2 cells in vitro (Flores-Villaseñor et al. 2012b). Also LFcin17–41, which is an extended variant of the LFcin17–30 was found to inhibit internalization of invasion-expressing *E. coli* (Longhi et al. 1994). Besides, LFcin17–41 was found to inhibit internalization of *Y. enterocolitcia* and *Y. pseudotuberculosis* in vitro (Di Biase et al. 2004). In our study however, LFcin17–30 showed no effect on adhesion and invasion of HeLa cells by the *Yersinia* spp. The additional 11 amino acids in LF17–41 force LFcin17-4 into a β-sheet conformation and appear to be crucial for this characteristic (Hwang et al. 1998). In contrast, LFcin17–30 conducts partially an α-helix conformation (Haney et al. 2012). Moreover, the parent protein bLF did not affect *Pseudomonas aeruginosa* adhesion to primary bronchial epithelium from a cystic fibrosis (CF) patient but significantly reduced the number of intracellular bacteria (Frioni et al. 2014). This bLF effect was attributed to a reduction of the intracellular bacterial survival. However, in our study the LFchimera inhibited invasion (Figs. 3, 4) while having no effect on the intracellular survival (data not shown).

Although this study primarily focused on the effect of the LFchimera on adhesion and invasion some speculations can be made on the molecular mechanisms of the inhibition of invasion. In case of *Yersinae* spp. the protein responsible for adhesion and invasion is invasin, an adhesive protein encoded by the *invA* gene on the bacterial chromosome (Atkinson and Williams 2016; Chauhan et al. 2016). Invasin is expressed at the bacterial surface and binds to cellular β1 integrins with a much higher affinity than natural extracellular matrix ligands, such as fibronectin (Tran Van Nhieu and Isberg 1993). Subsequently, invasin has the ability to evoke dimerization, allowing the clustering of β1 integrins on the interacting cell (Dersch and Isberg 1999), which then induces the formation of pseudopods to internalize bacteria (Gillenius and Urban 2015; Niemann et al. 2004). Based on the present finding that LFchimera inhibited bacterial invasion, but not adhesion, apparently mediated by an effect on the HeLa cells, it is hypothesized that the LFchimera specifically influenced the clustering of β1-integrins, thereby preventing downstream internalization.

Triggered by the cellular response of LFchimera on invasion the effect on proinflammatory cytokines like IL-8 an IL-6 was monitored. LFchimera induced stimulation of IL-8 expression by *Yersinia* infected cells and exhibited a direct effect on the stimulation of IL-6 expression, independent from *Yersinia* infection (Fig. 5). In this respect it is interesting that the level of IL-8 secreted by HeLa cells in response to invasion by *Y. entrocolitica*, was significantly lower with virulent strains than with non-virulent strains (Schulte et al. 1996, 2000). Of course further experimental data are needed to elucidate the underlying mechanism. The effects of bLF on the intracellular survival of bacterial pathogens and the differential modulation of the inflammatory response of epithelial models (Frioni et al. 2014) may be a lead but also shows that the mechanism is very complex and strongly depends on the system used (Kim et al. 2012; Sessa et al. 2017).

In conclusion, this research shows that LFchimera has the capacity to inhibit host-cell invasion by *Yersiniae* spp. and that this effect is host-cell mediated and not bacteria mediated. The mechanism might be linked to effect of invasion on β1 integrins but remains to be further investigated. Exposure of the host cells to both LFchimera and the bacterial strains elicits a pro-inflammatory cytokine release from the cells.
